# Supplementary figures and images for: Susceptibility of Female Mice to the Dietary Omega-3/Omega-6 Fatty-Acid Ratio: Effects on Adult Hippocampal Neurogenesis and Glia
Source: Int J Mol Sci. 2022 Mar 21;23(6):3399. doi: 10.3390/ijms23063399 (PMC8950413; doi:10.3390/ijms23063399)

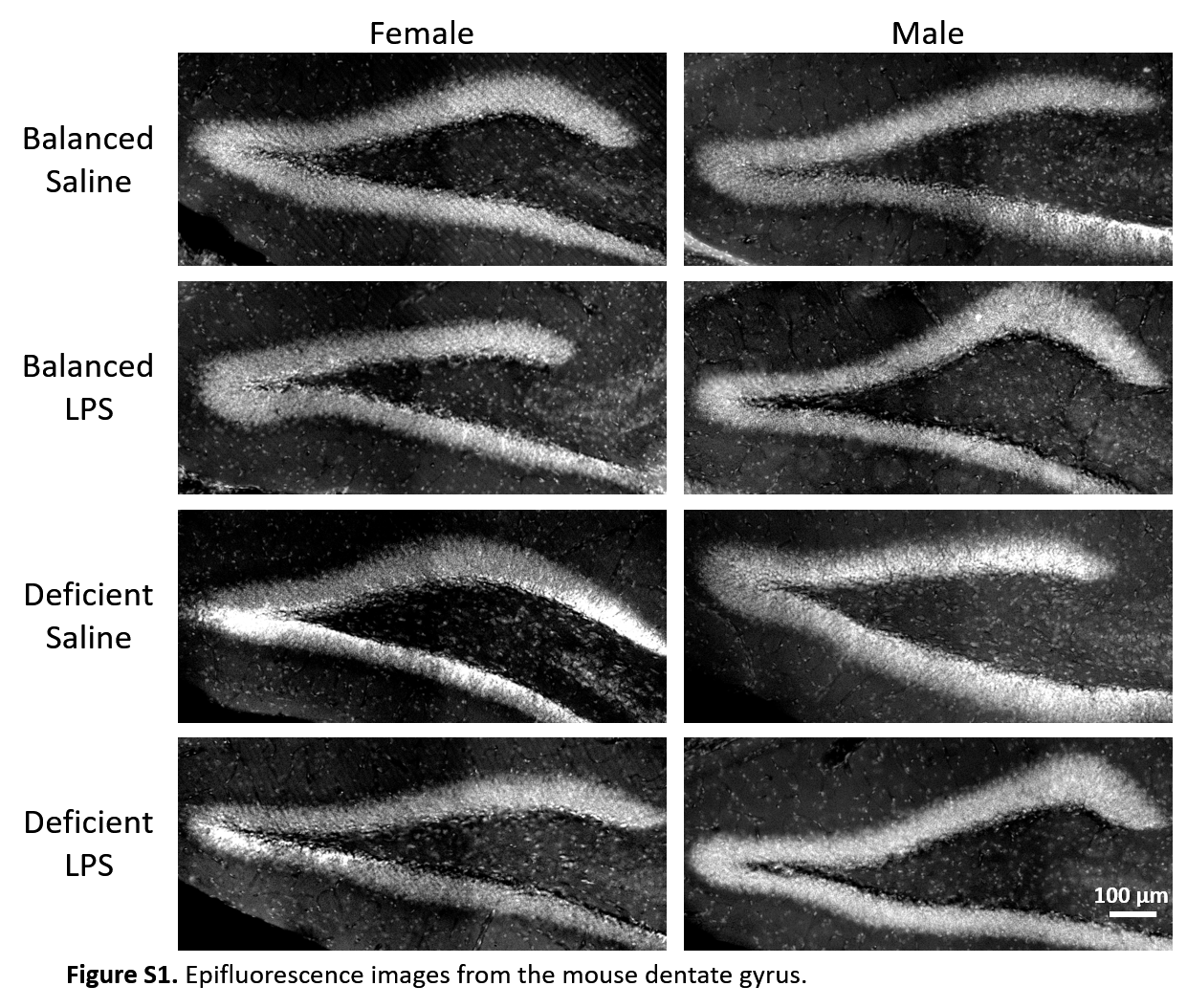

Supplement: Supplementary file 1 [file ijms-23-03399-s001.zip › Supplementary material/S1.png]

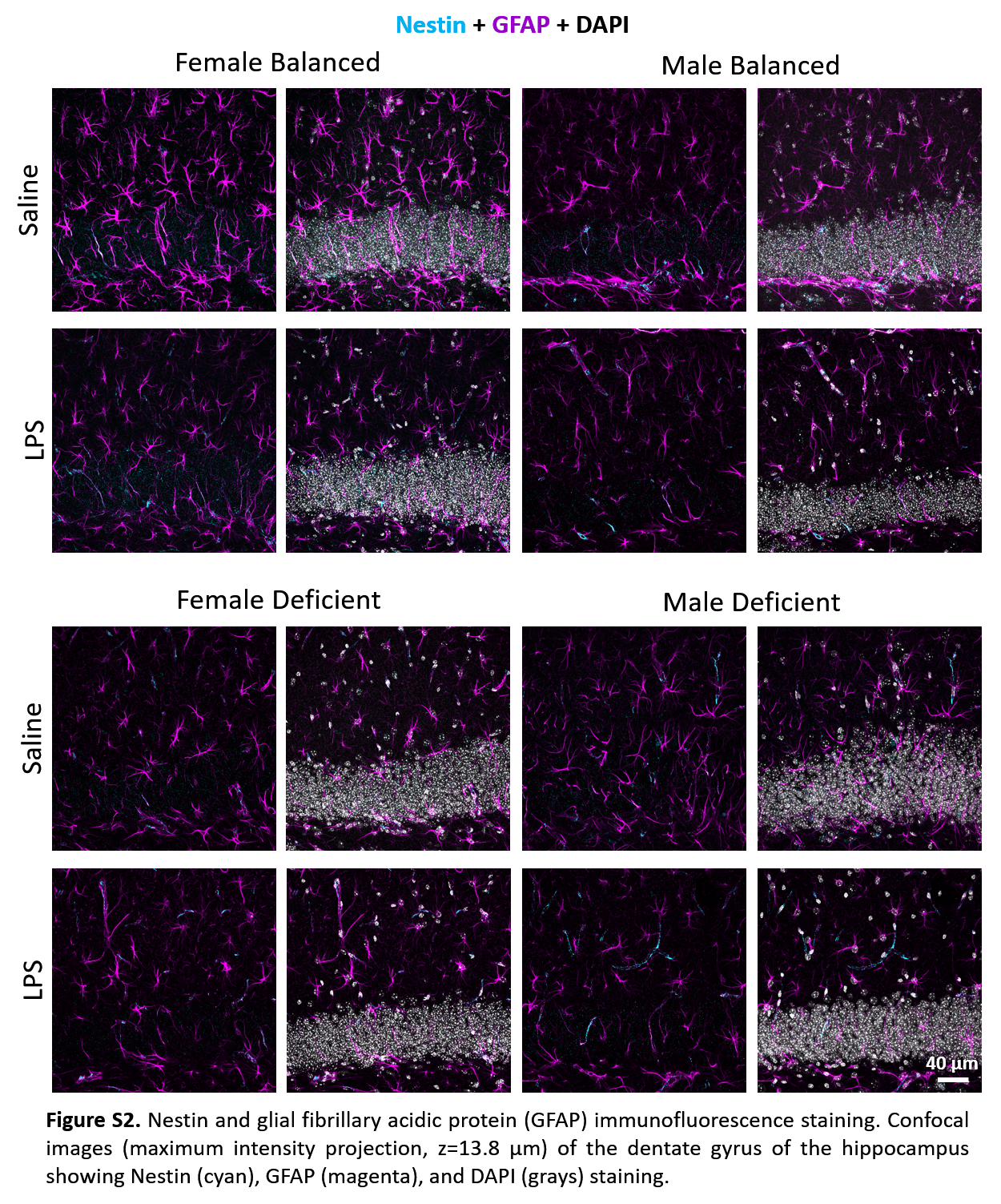

Supplement: Supplementary file 1 [file ijms-23-03399-s001.zip › Supplementary material/S2.png]

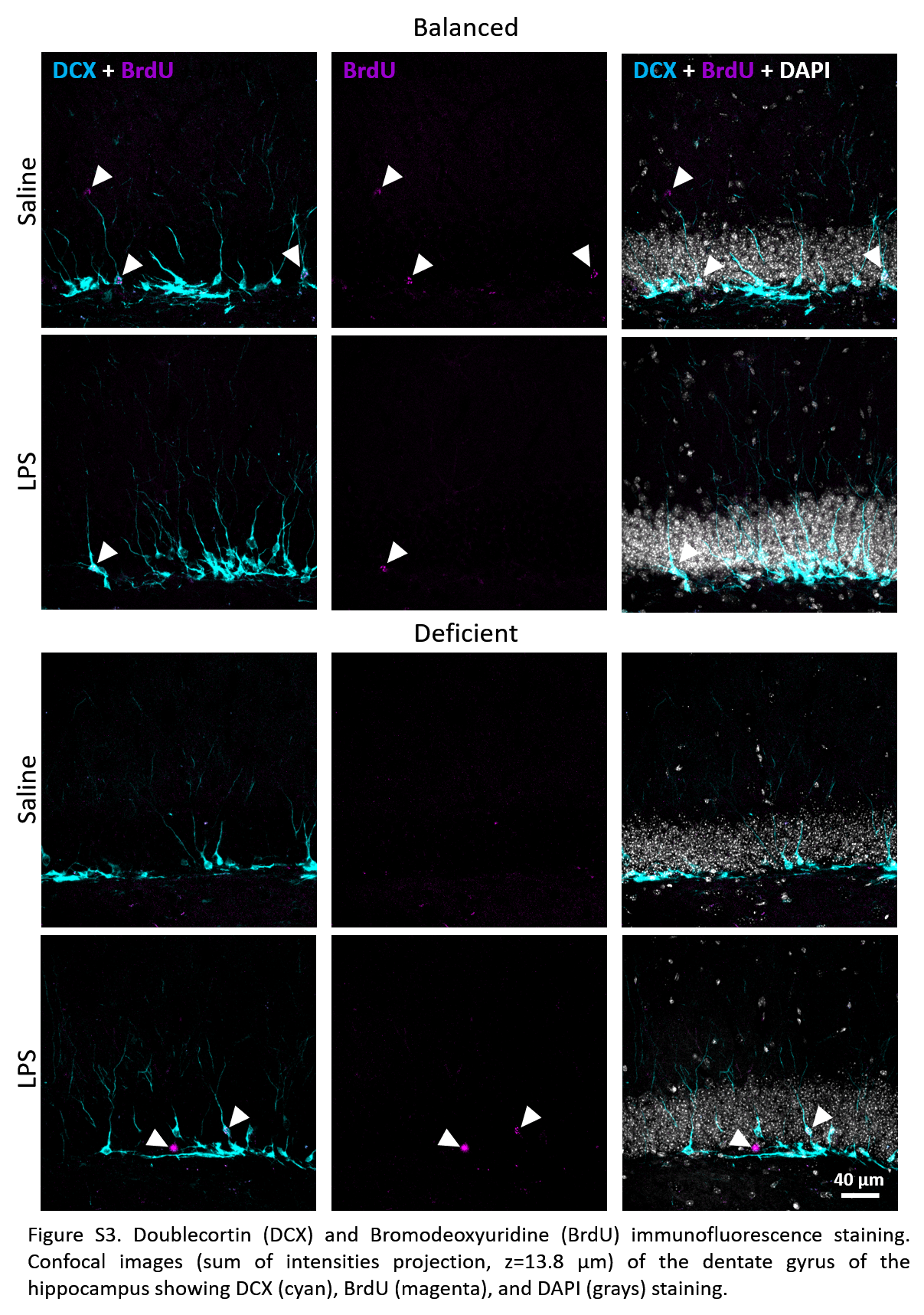

Supplement: Supplementary file 1 [file ijms-23-03399-s001.zip › Supplementary material/S3.png]

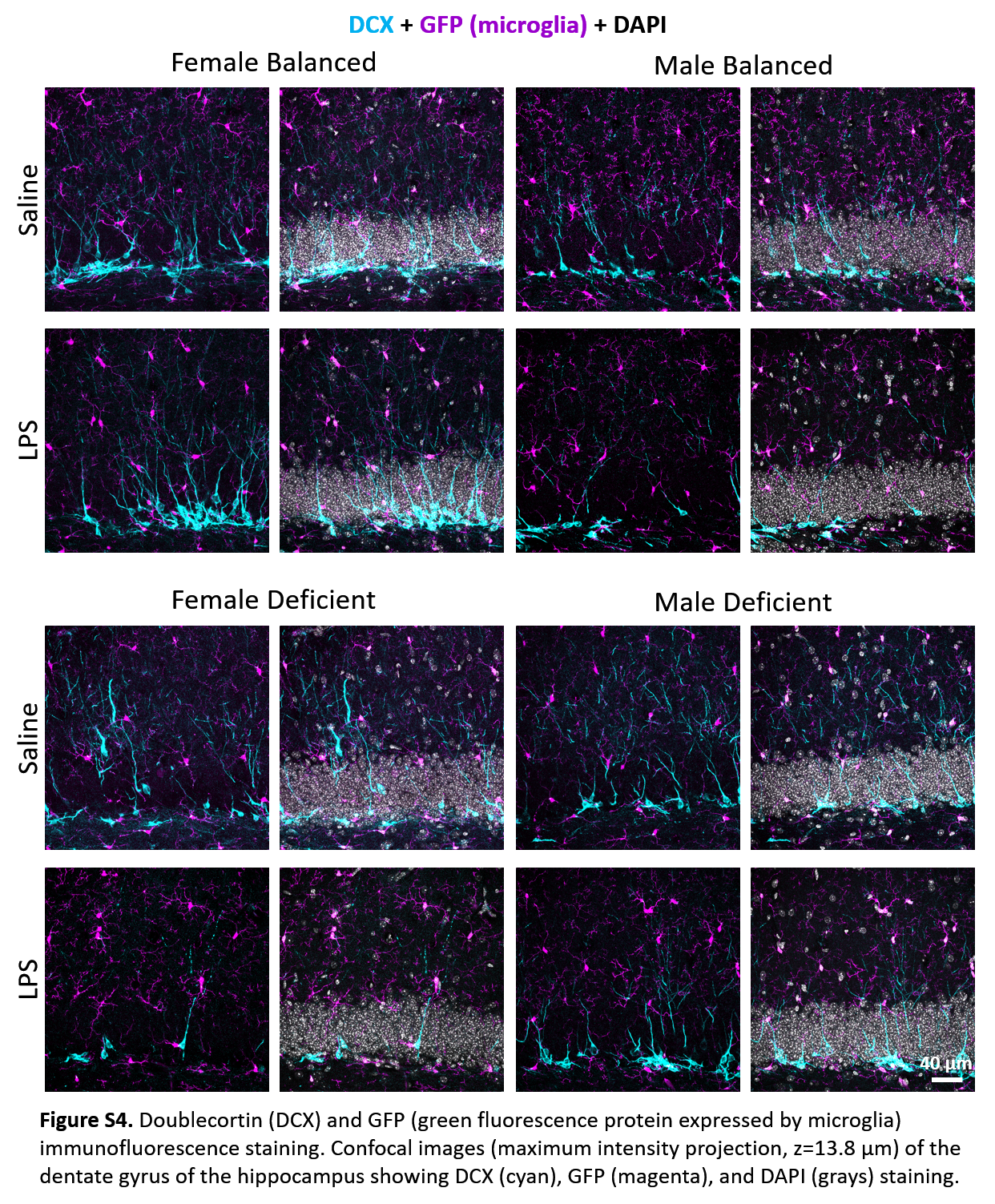

Supplement: Supplementary file 1 [file ijms-23-03399-s001.zip › Supplementary material/S4.png]
